# Responses of innate immune cells to group A Streptococcus

**DOI:** 10.3389/fcimb.2014.00140

**Published:** 2014-10-02

**Authors:** Christina Fieber, Pavel Kovarik

**Affiliations:** Max F. Perutz Laboratories, Department of Microbiology, Immunobiology and Genetics, University of ViennaVienna, Austria

**Keywords:** group A Streptococcus, *Streptococcus pyogenes*, innate immune response

## Abstract

Group A Streptococcus (GAS), also called *Streptococcus pyogenes*, is a Gram-positive beta-hemolytic human pathogen which causes a wide range of mostly self-limiting but also several life-threatening diseases. Innate immune responses are fundamental for defense against GAS, yet their activation by pattern recognition receptors (PRRs) and GAS-derived pathogen-associated molecular patterns (PAMPs) is incompletely understood. In recent years, the use of animal models together with the powerful tools of human molecular genetics began shedding light onto the molecular mechanisms of innate immune defense against GAS. The signaling adaptor MyD88 was found to play a key role in launching the immune response against GAS in both humans and mice, suggesting that PRRs of the Toll-like receptor (TLR) family are involved in sensing this pathogen. The specific TLRs and their ligands have yet to be identified. Following GAS recognition, induction of cytokines such as TNF and type I interferons (IFNs), leukocyte recruitment, phagocytosis, and the formation of neutrophil extracellular traps (NETs) have been recognized as key events in host defense. A comprehensive knowledge of these mechanisms is needed in order to understand their frequent failure against GAS immune evasion strategies.

## Introduction

The Gram-positive bacterium group A Streptococcus (GAS), also named *Streptococcus pyogenes*, causes a large variety of diseases ranging from mostly mild pharyngitis and impetigo to serious post-streptococcal sequelae. In extreme cases, GAS infections lead to invasive life-threatening diseases such as necrotizing fasciitis or streptococcal toxic shock syndrome. Severe invasive infections result in mortality exceeding 25%, and account for more than 650.000 cases each year (Carapetis et al., 2005; Johansson et al., 2010; Wessels, 2011). The ability of GAS to cause diseases with multiple clinical manifestations results from virulence factors which are variably expressed among GAS strains and from different host susceptibilities (Cunningham, 2000; Tart et al., 2007; Bessen, 2009; Cole et al., 2011; Lynskey et al., 2011).

**Figure 1:**
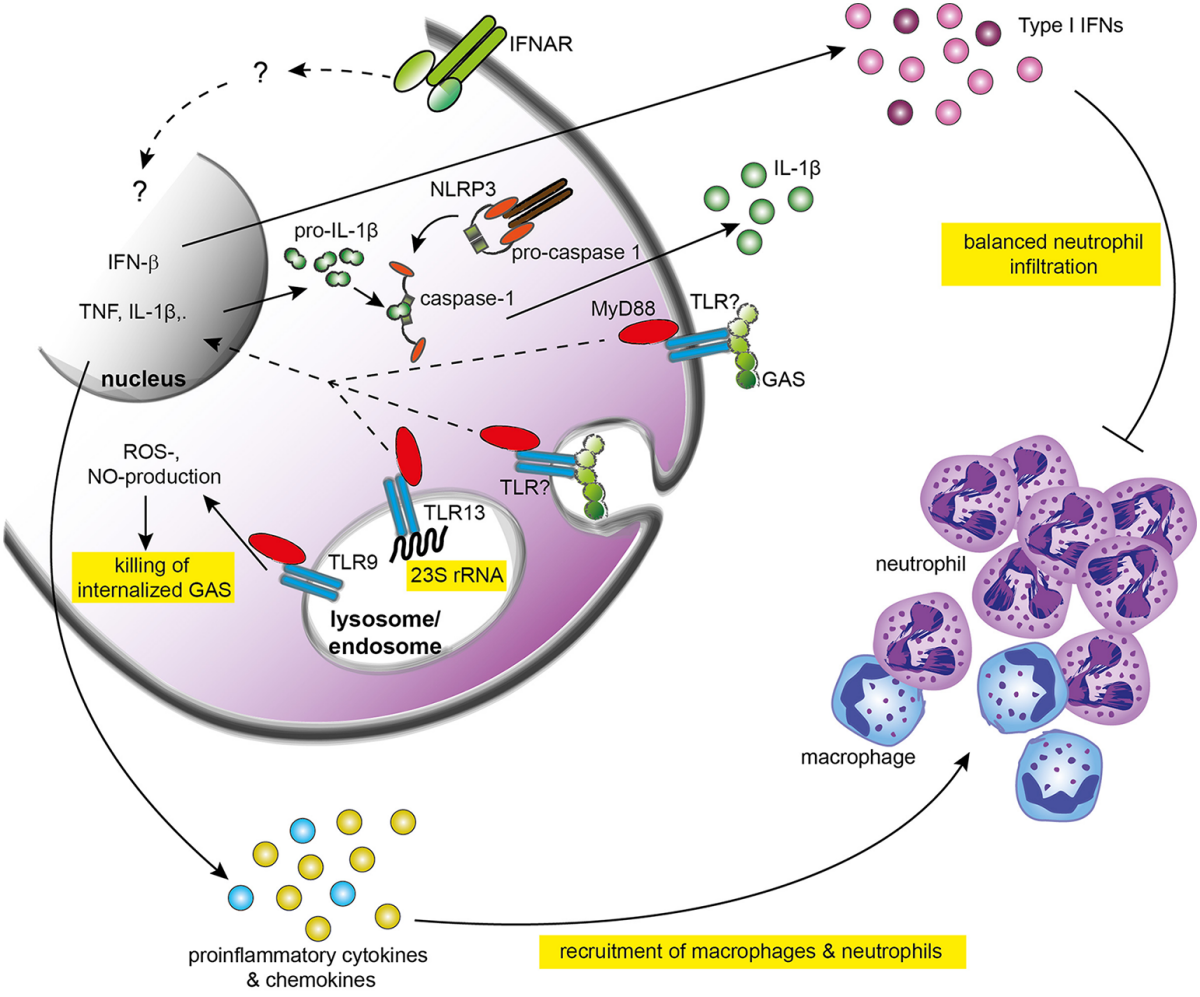
**Recognition of GAS and activation of innate immune responses**. GAS is recognized by yet not identified TLRs which signal through MyD88. The signaling cascade triggered by TLRs and MyD88 activates the expression of IFN-β and pro-inflammatory cytokines including TNF and IL-6. TNF promotes recruitment of macrophages to the infection site and resistance of mice against GAS. Type I IFN signaling elicited by IFN-β and other type I IFNs initiate so far poorly understood responses that culminate in balanced infiltration of neutrophils and protective immune responses against GAS. TLR13 recognizes bacterial 23S rRNA, including GAS rRNA, and might be involved in activation of innate immune responses. TLR9 facilitates killing of GAS by stimulating nitric oxide production. IL-1β is induced by GAS in a NLRP3-dependent manner but the physiological function of IL-1β remains unclear. GAS, group A Streptococcus; IFN, interferon; IFNAR, type I IFN receptor 1; IL-1β, interleukin 1β; NLRP3, NOD-like receptor family, pyrin domain-containing 3; NO, nitric oxide; ROS, reactive oxygen species; TLR, Toll-like receptor.

Upon GAS detection, the immune system launches a complex response which critically depends on the recruitment and activation of neutrophils, macrophages, and dendritic cells (DCs) (Goldmann et al., 2004; Loof et al., 2007; Zinkernagel et al., 2008; Mishalian et al., 2011). These processes are dependent on the activation of innate immune responses by interactions between pattern recognition receptors (PRRs) with GAS-derived pathogen-associated molecular patterns (PAMPs). Although advances in human genetics and new animal models have generated considerable progress in recent years, the individual components of these interactions and their precise roles in orchestrating the innate immunity remain incompletely understood (Figure 1). This review focuses on the molecular mechanisms of interactions between innate immune cells and GAS, and discusses their significance in the outcomes of an infection.

## Innate immune cells in defense against GAS

Despite limitations resulting from differences between the human and mouse immune systems, numerous studies have highlighted the value of mouse experiments for establishing the basic principles of innate immune defense against GAS. Mouse infection models were also instrumental for dissecting the roles for individual innate immune components in resistance against GAS infection.

One of the first studies addressing the contribution of specific innate immune cells to the early control of GAS infection employed a chemical, namely carrageenan, for depleting macrophages in mice (Goldmann et al., 2004). The study demonstrated the requirement for macrophages in limiting GAS dissemination and increasing mouse survival. Chemical inhibition of phagocytosis with gadolinium III chloride yielded a phenotype similar to chemical macrophage depletion, hence emphasizing the importance of phagocytosis for resistance to GAS. Due to the use of rather unspecific chemical inhibitors, these initial studies did not address the specific function of macrophages in the context of complex immune response. This limitation has been addressed in a recent study by Hanski and colleagues, who employed genetic means for macrophage depletion (Mishalian et al., 2011). Diphtheria toxin-mediated depletion of CCR2-positive macrophages resulted in an increased GAS dissemination and higher death rate. Interestingly, TNF-deficient mice failed to accumulate macrophages at the infection site whereas the infiltration of neutrophils was not impaired. These findings implicate that tissue-resident macrophages need a supportive activity of newly recruited macrophages for the establishment of protective defenses. Although the source remains to be determined, it is likely that macrophage-derived TNF acts in autocrine and paracrine ways to facilitate chemotactic relocation of macrophages to the site of infection. Alternatively, tissue-resident DCs, alone or in combination with macrophages, may be the critical source of this cytokine (Loof et al., 2008). Consistent with a substantial role for DCs, mice genetically depleted of CD11c^+^ DCs failed to control GAS dissemination. It remains to be elucidated whether the major function of DCs in host protection is the production of TNF or whether other DC-derived cytokines are also important (Loof et al., 2008). For example, DCs are a significant source of GAS-elicited IL-12 (Loof et al., 2007, 2008) which stimulates natural killer (NK) cells to generate interferon gamma (IFN-γ). Although a contribution of both IL-12 and IFN-γ to immune responses against GAS was reported, the specific function of these cytokines is currently not known (Metzger et al., 1995; Raeder et al., 2000). As IFN-γ is a major macrophage-activating cytokine (Nathan et al., 1983), the IFN-γ-generating DC/NK cell circuit might primarily serve to enhance macrophage antimicrobial functions. Interestingly, NK cells were reported to contribute to streptococcal toxic shock syndrome implicating that unrestricted stimulatory function of NK cells is harmful to the host (Goldmann et al., 2005a).

Many cytokines and chemokines produced by macrophages and DCs upon exposure to GAS, most notably IL-1β and CXCL1, are neutrophil recruitment factors (Harder et al., 2009; Kurupati et al., 2010). Exhaustion of bone marrow neutrophils and the resulting impaired infiltration of these cells to the site of infection increase the susceptibility to GAS (Navarini et al., 2009), implicating that there is a requirement for neutrophils in host protection. In agreement, antibody-mediated neutrophil depletion causes an avirulent GAS strain to become hypervirulent (Hidalgo-Grass et al., 2006). Neutrophils control bacterial infections largely through the production of antimicrobial peptides, reactive oxygen species (ROS) and, depending on the pathogen, neutrophil extracellular traps (NETs). GAS induces an extensive formation of NETs, which were found to prevent GAS from dissemination (Sumby et al., 2005; Buchanan et al., 2006; Lin et al., 2009). The ability to produce NET-like structures has also been reported for mast cells (Von Kockritz-Blickwede et al., 2008). Consistently, these immune cells are involved in efficient recruitment of leukocytes to the site of infection and clearance of GAS. Mast cell-deficient mice exhibit a decreased ability to contain GAS infection in part due to the lack of the mast cell-derived antimicrobial peptide cathelicidin (Di Nardo et al., 2008).

Although all major innate immune cells non-redundantly participate in host protection against GAS, more research is needed to define the specific roles for individual cell types, and to decipher the cross-talk between them. The role for the adaptive immune system in GAS infection is, as of yet, less well explored in mice as most GAS infection models address innate immunity. SCID mice, i.e., mice deficient in B and T cells, exhibit similar resistance against GAS as control mice suggesting a more profound role for the innate than the adaptive immune system (Medina et al., 2001; Goldmann et al., 2005b; Mishalian et al., 2011). Nonetheless, the importance of adaptive immunity might be underestimated, as humans lacking the signaling adaptor MyD88, which is required for activation of the innate immune response (see below), are highly susceptible to pyogenic infections during early life but less in the adulthood (Von Bernuth et al., 2008). This indicates that adaptive immune responses, which gradually develop during life, can to some extent substitute for the innate immune deficiency. The successful use of intravenous polyspecific immunoglobulins (IVIG) as adjuvant therapy corroborates the importance of adaptive immune mechanisms in protection against GAS (Johansson et al., 2010). In further support of this, recent studies by Cleary and colleagues uncovered a fundamental contribution of IL-17-producing Th17 cells to the clearance of GAS in a mouse model of pharyngitis (Wang et al., 2010; Dileepan et al., 2011). These Th17 cells developed only upon repeated immunization of mice implicating that these cells were antigen-specific. Although the precise nature of the host-protective Th17-dependent mechanism is not known, it can be assumed that the IL-17-mediated neutrophil expansion will enhance neutrophil presence in infected tissues. Interestingly, IL-17 can also be produced by the gammadelta T cells (γδ T cells) (Martin et al., 2009), which respond in an antigen-independent way to pathogens and link the adaptive and innate immune systems. Future studies should investigate whether IL-17 helps in the eradication of GAS in adult human patients deficient in the MyD88-dependent first line of defense (Von Bernuth et al., 2008).

## PRRs in GAS infection

Innate immune responses are triggered by interactions of PRRs with the cognate PAMPs (Medzhitov and Janeway, 2000; Takeuchi and Akira, 2010). PRR ligation by PAMP activates signaling cascades which lead to the production of cytokines and chemokines, and ultimately, to the recruitment of additional immune cells to the site of infection. TLRs are a membrane-bound subset of PRRs, which stimulate immune cells by activating the transcription factor NFκ B, but also the IRF family of transcription factors (Medzhitov et al., 1997; Kawai and Akira, 2010). TLRs signal mainly via the signaling adaptor MyD88 but also through TIR-domain-containing adapter-inducing interferon-β (TRIF) and TIR domain-containing adaptor protein (TIRAP)/MyD88 adaptor-like (Mal) (Medzhitov et al., 1998; Horng et al., 2001; Yamamoto et al., 2002; Oshiumi et al., 2003). TLRs are expressed on most innate immune cells as well as some adaptive immune cells. Epithelial and endothelial cells can also upregulate TLR expression upon stimulation (Kagnoff and Eckmann, 1997; Andonegui et al., 2003; Iwasaki and Medzhitov, 2004). Cytosolic PRRs include the RIG-I-, NOD-, and AIM2-like receptors (RLRs, NLRs, and ALRs, respectively) which recognize a variety of PAMPs and danger-associated molecular patterns (DAMPs), with various nucleic acids being the best characterized class of them (Franchi et al., 2008; Alnemri, 2010; Bauernfeind and Hornung, 2013; Ferrand and Ferrero, 2013). The family of nucleic acid-sensing cytosolic PRRs also includes the newly characterized DNA sensor cGAS (Ablasser et al., 2013; Li et al., 2013).

MyD88 is needed for launching a functional inflammatory response to GAS infection (Loof et al., 2008, 2010). MyD88 deficiency results in a dramatically decreased production by phagocytic cells of TNF, IL-6, IL-12, type I interferons (IFNs), and possibly most other cytokines and chemokines (Gratz et al., 2008, 2011; Loof et al., 2008, 2010) (Figure 1). Consequently, mice lacking MyD88 exhibit impaired recruitment of neutrophils to the site of infection and a higher susceptibility to GAS challenge (Loof et al., 2008, 2010). Consistently, TNF, one of the major pro-inflammatory cytokines generated downstream of TLR/MyD88 signaling, is required for the rapid recruitment of macrophages to the infected tissue and for prevention of invasion by GAS (Mishalian et al., 2011). Similarly, type I IFNs, which are induced in a largely MyD88-dependent manner, are critical for host defense as mice deficient in the type I IFN receptor 1 (IFNAR-1) exhibit higher susceptibility in a model of invasive GAS infection (Gratz et al., 2011) (Figure 1). An increased accumulation of neutrophils in the infected tissue of IFNAR-1-deficient mice suggests that other mechanisms than insufficient immune responses are responsible for the exacerbated GAS infection in the absence of type I IFN signaling. Despite the clear requirement for MyD88 in GAS-elicited production of key cytokines, the TLRs engaged by GAS upstream of MyD88 are not known. A number of studies demonstrated that primary innate immune cells derived from mice deficient in TLR1, TLR2, TLR4, TLR9, or TLR2/6 double-deficient, or TLR2/4/9 triple-deficient, were still able to produce inflammatory cytokines in response to GAS (Gratz et al., 2008; Loof et al., 2008, 2010). In particular, the absence of a substantial effect of TLR2 deficiency is surprising as this TLR is generally involved in the recognition of Gram-positive bacteria. It should be noted that these experiments mostly employed infection of cells rather than mice, so that the TLRs tested might still play a role in the context of a whole animal infection. In support of this, TLR9, although not required for cytokine induction *in vitro*, facilitates intracellular killing of GAS by inducing the production of reactive oxygen species and nitric oxide (NO) in macrophages (Zinkernagel et al., 2012). In contrast, mice lacking TLR2 appear to be only modestly more susceptible to GAS infection as the difference with control mice is not significant (Mishalian et al., 2011). Interestingly, similarly moderate increase in susceptibility was also observed in mice deficient in the monocyte-attracting CCL2 chemokine receptor CCR2 despite the importance of monocytes recruitment for resistance against GAS (Mishalian et al., 2011). Since the lack of CCR2 reduces the number of circulating monocytes by 80%, these observations clearly suggest that mice possess highly potent and possibly redundant macrophage activation pathways, which allow the establishment of a protective response against GAS also under conditions of greatly reduced macrophage numbers. Similar explanations might apply to the lack of a substantially higher susceptibility of TLR2-deleted mice.

GAS can trigger PRRs other than TLRs, as demonstrated by the induction of IL-1β in mouse macrophages (Harder et al., 2009). IL-1β production requires the NLRP3 inflammasome and depends on the expression of the GAS-derived cytolysin streptolysin O (SLO). Despite the strong induction of IL-1β by GAS, there is no evidence that this cytokine is required for a successful defense since NLRP3-deficient mice exhibit a similar susceptibility to intraperitoneal GAS infection as control animals (Harder et al., 2009). Thus, the GAS-specific function of IL-1β, one of the most potent inflammatory cytokines, remains to be identified.

GAS-derived PAMPs triggering MyD88-dependent or -independent responses are as enigmatic as the corresponding PRRs. Peptidoglycan purified from GAS can activate TLR2 (Schwandner et al., 1999), however, as mentioned above, no major contribution of TLR2 to responses elicited by live GAS can be observed. Possible explanations for these findings include a redundancy of TLR receptors or the masking of peptidoglycan in intact GAS by e.g., the hyaluronic acid capsule which is a critical immune evasion factor (Stollerman and Dale, 2008). In the latter scenario, peptidoglycan would become accessible only upon phagocytosis and the subsequent degradation of internalized GAS. The phagosome-specific configuration of TLRs may prevent substantial TLR2 responses yet allow other TLRs to be triggered by PAMPs distinct from TLR2 ligands. Such PAMPs include GAS-derived nucleic acids which act as PAMPs inducing IFN-β (a type I IFN) (Gratz et al., 2011). Interestingly, GAS RNA triggers IFN-β production in a MyD88-dependent manner in DCs, but it fails inducing this cytokine in macrophages, suggesting that more attention needs to be paid to cell type-specific PRR-PAMP interactions.

Together, despite the fundamental importance of the signaling adaptor MyD88 for protection against GAS infection, the upstream TLRs as well as the precise nature of their ligands remain to be elucidated. Similarly, the mechanism of the NLRP3 inflammasome activation requires further investigations. Future studies should also address the *in vivo* role for the recently characterized TLR13, a MyD88-dependent receptor, which specifically recognizes bacterial 23S rRNA, including GAS rRNA (Hidmark et al., 2012; Li and Chen, 2012; Oldenburg et al., 2012). As TLR13 is expressed in mice but not humans, it is intriguing whether any of the human TLRs can substitute for the TLR13-mediated responses.

## Evasion of innate immune responses

**Figure 2:**
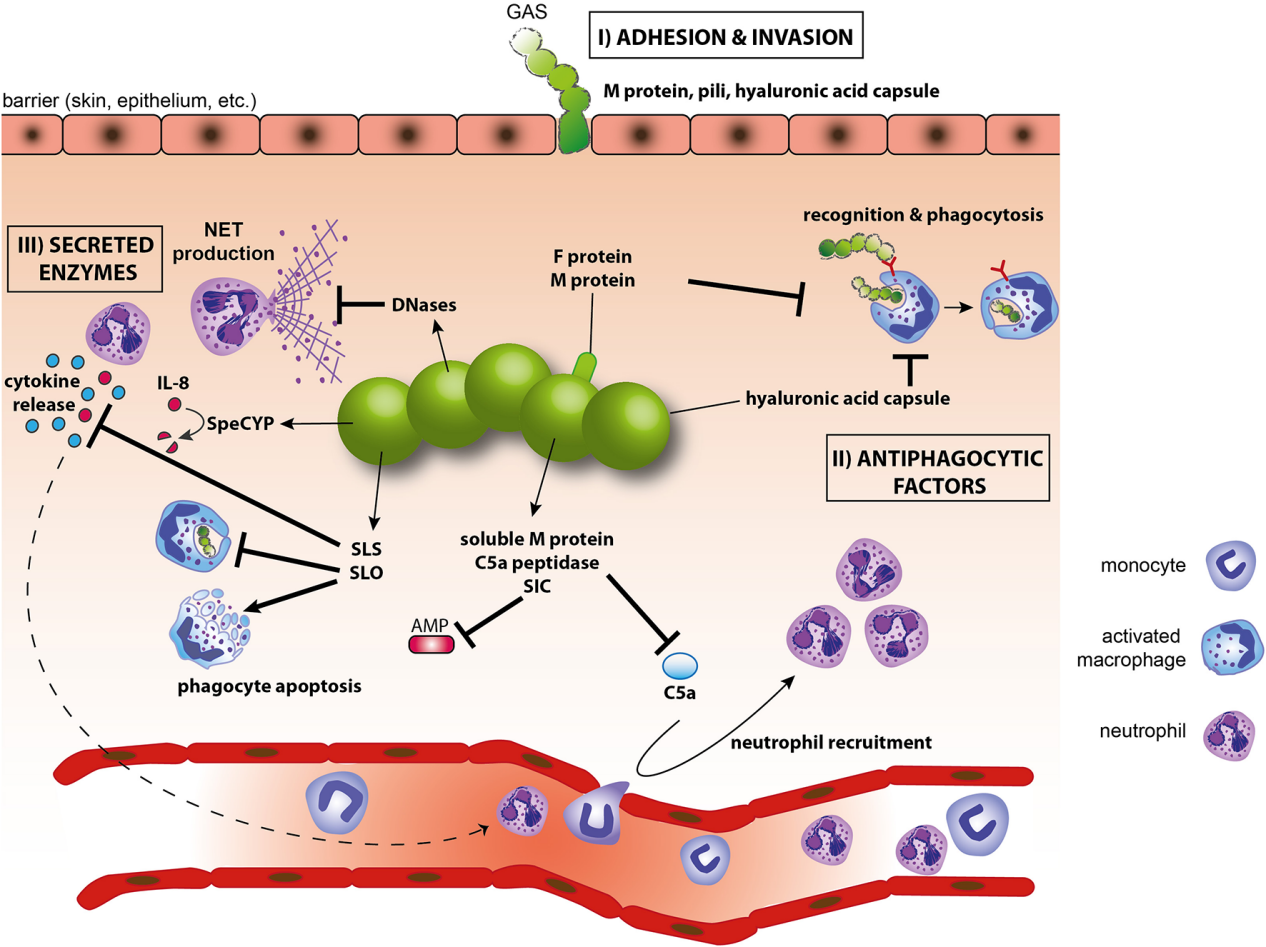
**Evasion of innate immunity by GAS**. (I) M protein, F proteins of pili and the hyaluronic acid capsule are involved in adhesion and/or invasion; (II) SLO, hyaluronic acid capsule and M-proteins inhibit phagocytosis and help avoid killing of GAS within phagolysosomes; (III) Secreted GAS factors inhibit complement activation and antimicrobial peptides (C5a peptidase, SIC), prevent phagocyte recruitment (C5a peptidase), induce apoptosis of phagocytes (SLO), interfere with cytokines or cytokine production (SLS, SpeCYP), destroy NETs (DNases). AMP, antimicrobial peptide; NET, neutrophil extracellular trap; SIC, streptococcal inhibitor of complement; SLO, streptolysin O; SLS, streptolysin S.

GAS possesses a large variety of strategies to evade the host immune response (Figure 2). To avoid the first line of defense, i.e., phagocytic cells, GAS employs antiphagocytic virulence factors such as the M1 protein and the hyaluronic acid capsule (Moses et al., 1997). Phagocytosis is an efficient host defense mechanism as the vast majority of internalized GAS is rapidly killed. Phagocytosed GAS can increase its survival by the expression of the pore-forming cytolysin SLO which inhibits the transport of GAS to lysosomes and facilitates escape of GAS from lysosomes (Nakagawa et al., 2004; Hakansson et al., 2005). SLO has a more general function in immune evasion since it also induces cell death in macrophages and neutrophils (Goldmann et al., 2009; Timmer et al., 2009). GAS expresses yet another pore-forming toxin, the cytolysin SLS, which inhibits neutrophil recruitment to the site of infection by blocking the production of chemotactic signals (Lin et al., 2009). Neutrophils are also targeted by the prophage-encoded nucleases Sda1 and SpnA. These secreted DNases attenuate the host antimicrobial mechanisms by degrading NETs and liberating the entrapped GAS (Sumby et al., 2005; Buchanan et al., 2006; Walker et al., 2007; Chang et al., 2011). In addition to degrading NETs, GAS can dissolve blood clots via the secreted GAS protease streptokinase (Ska). Ska activates plasminogen by converting it into plasmin which is deposited on the cell surface of GAS and helps degrading fibrin clots and promotes GAS dissemination (Sun et al., 2004). GAS secreted proteases also play a key role in blunting chemokine activities. The proteinase SpeCYP interferes with neutrophil recruitment by cleaving and inactivating the neutrophil chemoattractants IL-8 in humans, as well as CXCL1 and CXCL2 in mice (Edwards et al., 2005; Hidalgo-Grass et al., 2006). Another immune evasion strategy is the blockage of the complement system. GAS impedes complement deposition by cleaving C5a, an important inducer of neutrophil recruitment, by the C5a peptidase as well as the anchorless surface dehydrogenase (Cleary et al., 1992; Terao et al., 2006). Streptococcal inhibitor of complement (SIC) inhibits complement-mediated formation of the membrane attack complex (MAC) and decreases antimicrobial peptide production by neutrophils (Akesson et al., 1996; Hoe et al., 2002; Frick et al., 2003). The M1 protein can bind the host Factor H, a potent inhibitor of complement deposition and phagocytosis. Surprisingly, a recent study has demonstrated that binding of Factor H to M1 does not interfere with phagocytosis (Gustafsson et al., 2013) implicating that the function of some virulence factors may not be correctly annotated. A broader use of humanized mice will further improve the functional characterization of GAS evasion mechanisms, as exemplified by mice expressing the human plasminogen (Sun et al., 2004).

## Conclusions

The wealth of *in vitro* data, infection studies and human genetics demonstrate a crucial role of the innate immune system in protection against GAS infection. Yet, our knowledge of specific interactions between PRRs and PAMPs is surprisingly limited. This knowledge is needed to design new strategies for treatments of severe GAS infections. Such strategies should include the development of PRR agonists and antagonists, which might help adjusting the immune responses so that they precisely match the extent and tissue-specific features of the bacterial challenge. A better understanding of PRR-PAMP interactions could also reveal additional mechanisms underlying the highly variable susceptibility to GAS infections among humans. Future studies should employ more sophisticated animal models, including conditional and/or inducible gene ablations and multiple knockouts to address tissue specificity and redundancy in responses to GAS. The use of CRISPR-Cas9-mediated genome editing, which was first described using GAS as a CRISPR model (Jinek et al., 2012), should facilitate the development of new cell culture models, particularly in the human system. As GAS is a human-specific pathogen, the combination of mouse models with cell-based experiments in the human system together with molecular epidemiology studies are critical for filling up the blank spots on the map of immune responses to GAS.

## Author contributions

Christina Fieber and Pavel Kovarik wrote the manuscript.

### Conflict of interest statement

The authors declare that the research was conducted in the absence of any commercial or financial relationships that could be construed as a potential conflict of interest.
